# Heart Rate Variability-Guided Training for Enhancing Cardiac-Vagal Modulation, Aerobic Fitness, and Endurance Performance: A Methodological Systematic Review with Meta-Analysis

**DOI:** 10.3390/ijerph181910299

**Published:** 2021-09-29

**Authors:** Agustín Manresa-Rocamora, José Manuel Sarabia, Alejandro Javaloyes, Andrew A. Flatt, Manuel Moya-Ramón

**Affiliations:** 1 Sports Research Centre, Department of Sport Sciences, Miguel Hernández University of Elche, 03202 Elche, Spain; amanresa@umh.es (A.M.-R.); jsarabia@umh.es (J.M.S.); ajavaloyes@umh.es (A.J.); 2 Institute for Health and Biomedical Research (ISABIAL Foundation), Miguel Hernandez University, 03550 Alicante, Spain; 3 Department of Health Sciences and Kinesiology, Georgia Southern University (Armstrong Campus), Savannah, GA 31419, USA; aflatt@georgiasouthern.edu

**Keywords:** autonomic nervous system, parasympathetic activity, heart rate recovery, resting heart rate, cardiorespiratory fitness

## Abstract

Purpose: This systematic review with meta-analysis was conducted to establish whether heart rate variability (HRV)-guided training enhances cardiac-vagal modulation, aerobic fitness, or endurance performance to a greater extent than predefined training while accounting for methodological factors. Methods: We searched Web of Science Core Collection, Pubmed, and Embase databases up to October 2020. A random-effects model of standardized mean difference (SMD) was estimated for each outcome measure. Chi-square and the I^2^ index were used to evaluate the degree of homogeneity. Results: Accounting for methodological factors, HRV-guided training was superior for enhancing vagal-related HRV indices (SMD_+_ = 0.50 (95% confidence interval (CI) = 0.09, 0.91)), but not resting HR (SMD_+_ = 0.04 (95% CI = −0.34, 0.43)). Consistently small but non-significant (*p* > 0.05) SMDs in favor of HRV-guided training were observed for enhancing maximal aerobic capacity (SMD_+_ = 0.20 (95% CI = −0.07, 0.47)), aerobic capacity at second ventilatory threshold (SMD_+_ = 0.26 (95% CI = −0.05, 0.57)), and endurance performance (SMD_+_ = 0.20 (95% CI = −0.09, 0.48)), versus predefined training. No heterogeneity was found for any of the analyzed aerobic fitness and endurance performance outcomes. Conclusion: Best methodological practices pertaining to HRV index selection, recording position, and approaches for establishing baseline reference values and daily changes (i.e., fixed or rolling HRV averages) require further study. HRV-guided training may be more effective than predefined training for maintaining and improving vagal-mediated HRV, with less likelihood of negative responses. However, if HRV-guided training is superior to predefined training for producing group-level improvements in fitness and performance, current data suggest it is only by a small margin.

## 1. Introduction

Habitual cardiorespiratory endurance exercise improves a variety of markers related to human health and performance [1]. Exercise programs that efficiently stimulate adaptations are therefore of interest to general, clinical, and athletic populations. Traditional exercise prescription methodology involves predefined program parameters in which the intensity, volume, frequency, and timing of training are scheduled in advance. Several predefined training models have been implemented to improve indices of fitness and performance in various populations [2,3]. Though group-level improvements in fitness-related outcomes support predefined training, responses at the individual level are mixed [4]. For instance, Bouchard, An, Rice, Skinner, Wilmore, Gagnon, Pérusse, Leon, Rao [5] reported an average increase in maximal oxygen uptake ($\dot{V}$O_2_ max) of 384 ± 202 mL∙min^−1^ after a standardized 20-week training program in 720 healthy subjects. However, individual responses ranged from decrements of 100 mL∙min^−1^ in some participants to increments of 1000 mL∙min^−1^ in others. Thus, individualized exercise prescription that modifies intensity, volume, and timing of exercise according to the evolving status of the participant may increase the effectiveness and efficiency of exercise training [6].

Cardiac-autonomic functioning, as indexed by vagal-mediated heart rate (HR) variability (HRV) indices (i.e., the root-mean-square difference of successive normal R-R intervals (RMSSD), the high frequency (HF), and the standard deviation of the instantaneous beat-to-beat R-R interval variability (SD_1_)) [7], is a non-invasive marker of acute and chronic adaptation to endurance exercise. In the short-term (e.g., within 48 h after exercise), recovery of HRV to baseline is thought to coincide with restoration of thermoregulatory, metabolic, hemodynamic, and fluid-balance related processes that are disturbed by physical exertion [8]. In the long-term (e.g., weeks to months), HRV profiles that reflect higher and/or more stable resting values have been associated with greater improvements in post-intervention fitness outcomes among sedentary [9], moderately-trained [9,10], highly-trained [11,12,13], and clinical populations [14,15,16]. Recent experiments have compared predefined training versus HRV-guided training, in which high intensity exercise is prescribed when resting HRV is within or above baseline ranges and low intensity exercise (or passive rest) is prescribed when values are suppressed. Some key findings favoring HRV-guided training include similar or greater improvements in selected fitness outcomes despite fewer high intensity sessions, less heterogeneity in fitness changes [17,18], and effectiveness in a variety of populations [17,18,19,20,21,22,23,24].

Recent reviews have aimed to consolidate available findings. Granero-Gallegos, González-Quílez, Plews, Carrasco-Poyatos [25] reported that HRV-guided training had a significantly greater effect on $\dot{V}$O_2_ max versus predefined training. However, this meta-analysis included the training group (i.e., HRV-guided training and predefined training) as the analysis unit. Therefore, within-group effect sizes (ESs), which exhibit lower internal validity than between-group ESs [26], were estimated. Moreover, these results should be interpreted with caution since testing for subgroup comparisons based on the training prescription method used was not performed. Medellin Ruiz, Rubio-Arias, Clemente-Suarez, Ramos-Campo [27] also compared HRV-guided training to predefined training for improving aerobic fitness and performance (i.e., $\dot{V}$O_2_ max and maximal power output) in endurance-trained athletes and sedentary subjects and reported no differences between training prescription methods. Nevertheless, heterogeneity analyses to test the influence of methodological approaches and/or individual differences were not performed. Finally, Düking, Zinner, Reed, Holmberg, Sperlich [28] carried out a systematic review on the effectiveness of HRV-guided training and predefined training in healthy runners. The authors reported that both training prescription methods induce physiological adaptations, with effects of HRV-guided training tending to be greater. Thus, collective findings are inconclusive. 

Various methodological approaches have been applied in HRV-guided training interventions that may influence outcomes and may possibly explain the lack of consensus in recent reviews [25,27,28]. Differences in HRV assessment (e.g., body position, pre-recording stabilization period, measurement duration, selection of the vagal-related HRV index, and respiration rate) and the criterion to modify training (e.g., use of single or average HRV values, and static or rolling baseline reference ranges) may influence HRV values and, consequently, training prescription. Additionally, the training status of the participants may influence both HRV and the effectiveness of the training program [29]. Highly trained individuals have less room for improvement and a greater tolerance for training stress than recreationally active and sedentary populations. Thus, a more thorough consolidation of the original research that accounts for the aforementioned methodological factors is needed.

Aerobic fitness and performance have been the primary outcomes of interest in recent reviews [25,27,28]. Whether post-intervention changes in markers of cardiac-parasympathetic modulation vary as a function of prescription methodology is unclear. Resting HR and HRV, as well as post-exercise HR recovery (HRR) are various markers of vagal activity, each of which are independent predictors of cardiovascular morbidity and mortality [30]. The ubiquity of mobile devices capable of tracking resting and exercise-related HR metrics has generated widespread interest in these parameters [31], possibly because they are modifiable by lifestyle behaviors [32,33]. HRV in particular exhibits considerable versatility in informing on health and wellbeing [32,33,34], longevity [35], fitness, and performance [36,37,38,39,40]. Thus, practical and effective interventions that improve HRV are of growing and universal interest [41]. Modification of exercise based on daily HRV is now accessible to the masses, but its efficacy for improving HRV requires clarification. 

A comprehensive investigation into the effectiveness of individualized endurance exercise based on daily HRV may be used to guide best practices for future research and inform applied implementation. Therefore, this systematic review with meta-analysis was conducted to establish whether HRV-guided training enhances cardiac-vagal modulation or aerobic fitness and performance to a greater extent than predefined training while accounting for methodological factors.

## 2. Methods

We conducted and reported a systematic review of the literature and a meta-analysis following the Preferred Reporting Items for Systematic Reviews and Meta-analyses (PRISMA) guidelines [42]. The systematic review and meta-analysis protocol were prospectively registered in the PROSPERO database (CRD42020218995).

### 2.1. Data Search and Sources

Potential studies were identified via a comprehensive strategy. A systematic search was performed in the Web of Science Core Collection, PubMed, and Embase databases from inception to October 2020 using free-text terms based on the PIC (participants, interventions, and comparisons) strategy. Language restrictions were not applied during this phase. The electronic search of individual databases was adapted as necessary (the full search strategy is depicted in the supplementary materials, Section S1). Moreover, the reference lists of previous reviews and full-text articles were manually checked to assess for eligibility. Conference proceedings were also searched on the Web of Sciences Core Collection database. Authors of selected studies were contacted via e-mail in an attempt to identify unpublished or ongoing studies that fulfilled our selection criteria. These search strategies were used to minimize the risk of publication bias.

### 2.2. Study Selection

Eligibility criteria were established according to the PICOS (participants, intervention, comparison, outcomes, and study design) guideline: (a) sedentary healthy people, physically active, and endurance-trained athletes, regardless of training status or sex (participants); (b) endurance training prescription in the experimental group based on changes in vagal-related HRV indices (intervention); (c) predefined endurance training prescription in the control group (comparison); (d) cardiac-vagal modulation (i.e., vagal-related HRV indices, HRR, and/or resting HR), aerobic fitness parameters (i.e., $\dot{V}$O_2_ max, maximal aerobic capacity, aerobic capacity at second ventilatory threshold (VT2), and/or aerobic capacity at first ventilatory threshold (VT1)), and/or endurance performance changes after the intervention (outcomes); and (e) randomized and non-randomized controlled trials (study design) written in English or Spanish. 

### 2.3. Data Extraction, Coding Study Characteristics, and Potential Moderator Variables

The following information was extracted from included studies: (a) study characteristics (publication year, country, study design (randomized or non-randomized), and journal); (b) baseline participant characteristics (sample size, sex (male, female, or mixed sample), age, $\dot{V}$O_2_ max, weight, athletic status (sedentary, physically active, recreational, or endurance-trained athletes), and sport (if applicable)); (c) exercise characteristics (training mode (endurance training or combined endurance and strength training), intervention length, and predefined training characteristics); (d) methodological approach characteristics (vagal-related HRV index (RMSSD, HF, or SD_1_), power spectral density (PSD) method (if applicable), HRV value (single or averaged), number of average HRV values (if applicable), time of the day, device used, body position (sitting, standing, and supine), measurement length, breathing control, smallest worthwhile change (SWC) or reference criterion (fixed or moving), number of average values (if applicable), and criteria for modifying training in the HRV-guided training group). 

### 2.4. Risk of Bias

The Cochrane Collaboration’s core risk of bias tool was uses to assess risk of selection, detection, attrition, and reporting bias, which were classified as high, unclear, or low risk of bias [43].

Two authors (AM and AJ) performed the study selection, data extraction, and risk of bias assessment. Disagreements were settled by consensus and, when consensus was not achieved, a third author (JMS) assessed the study or information to reach an agreement.

### 2.5. Computation of Effect Size and Statistical Analyses

The standardized mean difference (SMD) was used as the ES index to assess changes in cardiac-vagal modulation, aerobic fitness parameters, and endurance performance after the intervention. The SMD was calculated by subtracting the mean change in the outcome variables for the HRV-guided training group from the mean change for the predefined training group divided by the pooled standard deviation (SD) at baseline, corrected by a factor for small samples. SMD positive values indicated that it was favorable to HRV-guided training. In multiple-intervention studies with a shared predefined training group, the sample size in the predefined group was split-up [44], allowing us to include several analysis units from the same study. Separate analyses were performed for each SMD index according to the outcome measure when it was reported for at least three analysis units to avoid statistical dependence. A random-effects model was applied for each meta-analysis in which the weighting factor was the inverse variance, defined as the sum of the within-study and the between-studies variance. A conservative value of 0.7 previously proposed by Rosenthal [45] was used to calculate the variance of each study when the studies did not report the correlations between pre- and post-intervention measures. The analysis comprised calculating the mean ES with its 95% confidence interval (CI), a heterogeneity statistical test, chi-square, and the *I*^2^ index to evaluate the degree of homogeneity of the ESs around the average effect. The magnitude of the SMD was classified as trivial (<0.20), small (0.20–0.59), moderate (0.60–1.19), large (1.20–1.99), or very large (≥2.00) [46]. We considered a statistically significant effect when *p* ≤ 0.05. Heterogeneity was classified as low, moderate, or high at 25%, 50%, and 75%, respectively. In cases of substantial heterogeneity (chi-square test statistically significant and/or *I*^2^ index > 50%), moderator variables analyses were performed by assessing the relationship between the ESs and the potential categorical and continuous potential moderator variables using subgroup analysis and simple meta-regressions, respectively. All analyses were carried out using weighted least squares and assuming mixed-effects models. In case of substantial heterogeneity in vagal-related HRV results, tests for subgroup comparisons were performed based on the vagal-related HRV index (i.e., RMSSD, HF, and SD_1_) and the HRV value (i.e., single HRV value and averaged HRV value) to test the influence of methodological factors. For subgroup comparisons based on the vagal-related HRV index, RMSSD and SD_1_ were considered the same index (RMSSD/SD_1_), as previously reported [47]. In cases of substantial heterogeneity regardless of the outcome measure, the influence of participant and methodological approach characteristics on our findings were also investigated. Publication bias analyses were performed using a funnel plot with the trim-and-fill method for imputing possible missing ESs [48,49]. Finally, sensitivity analyses were performed to assess the influence of any individual study by removing each study and performing all analyses. Statistical procedures were performed using STATA software (version 16.0; Stata Corp LLC, College Station, TX, USA). For articles that did not report methodological information (e.g., single or averaged HRV values) or outcome data (i.e., mean or SD), authors were contacted via e-mail to obtain this information.

## 3. Results

### 3.1. Study Selection

**Figure 1:**
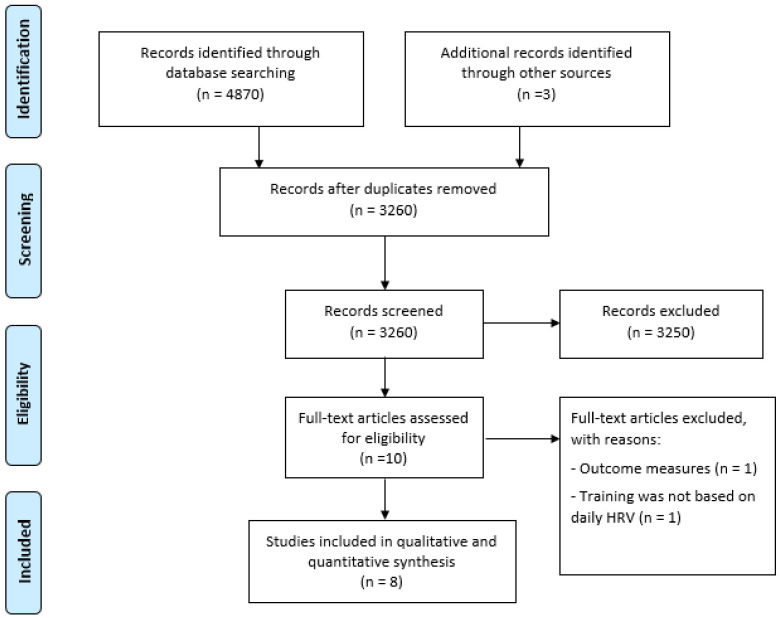
Flow chart of the systematic review process.

From a total of 3260 studies after removing duplicates, 10 were eligible for full text analysis [17,18,19,20,21,22,23,24,50,51], of which we excluded two studies from qualitative and quantitative synthesis as follows: based on the same sample and other outcome measures reported (*n* = 1) [50] and training not guided by daily HRV values (*n* = 1) [51]. Out of all the selected studies, Kiviniemi, Hautala, Kinnunen, Nissilä, Virtanen, Karjalainen, Tulppo [21] included three HRV-guided training groups and two predefined training groups, allowing us to include three analysis units. Therefore, a total of 10 analysis units were included in the final qualitative and quantitative synthesis. Although we attempted to locate unpublished studies, all the selected studies had been published in peer-reviewed journals. A Preferred Reporting Items for Systematic Reviews and Meta-analysis flow-chart of our literature search and selection is presented in Figure 1.

### 3.2. Study Characteristics

Study and participant characteristics are summarized in Table 1. The eight included studies are from four countries and were published between 2007 and 2020. Seven studies (88%) were randomized trials and one (12%) was a non-randomized trial [17]. In total, there were 199 participants (106 participants allocated to the HRV-guided training group and 93 in the predefined training group) with a mean ± SD age of 31.8 ± 4.8 years (min-max: 22.5–38.5 years), of which, 120 were males and 79 were females. Out of the 10 included analysis units, five (50%) were composed exclusively of male participants, three (30%) by female participants, and two (20%) used a mixed sample. Analysis unit sample size at pre-intervention varied from 14 to 40 participants. Based on the authors sample description, one analysis unit (10%) was composed of sedentary participants [19], three (30%) included physically active adults [21], three (30%) recruited recreationally trained athletes [22,23,24], and three (30%) included well-trained [17,20] and high-level athletes [18]. Out of all the analysis units composed of athletes, two were runners [22,24], two cyclists [17,20], one cross-country and nordic-skiers [18], while one study reported that endurance athletes were included [23]. The average ± SD weight and $\dot{V}$O_2_ max at pre-intervention were 71.7 ± 7.1 kg (min–max: 62.1–81.5 kg) and 51.3 ± 9.8 mL·kg^−1^·min^−1^ (min–max: 35.5–65.2 mL·kg^−1^·min^−1^), respectively. One study did not report participant weight [24] and another one did not assess $\dot{V}$O_2_ max [19].

Intervention and methodological approach characteristics are reported in Table 2. Five studies (62.5%) performed the intervention based on endurance training [17,19,20,21,22], two (25%) based on combined endurance and strength training [23,24] and one (12.5%) did not report this information [18]. The intervention length ranged from 2 to 8 weeks. Seven studies (87.5%) carried out daily HRV assessments in the morning after awakening [17,18,20,21,22,23,24] and one (12.5%) performed HRV measurements in the afternoon/evening before performing training sessions [19]. Seven studies (87.5%) explicitly reported that a stabilization period was performed before capturing HRV, ranging from 30 s to 5 min. Three studies (37.5%) carried out daily HRV assessments in the standing position, four (50%) in the supine position, and one (12.5%) in supine and standing positions. The assessment length ranged from 1 to 5 min. Three studies explicitly reported that participants were allowed to breathe spontaneously through HRV assessments [19,21,24], while the remaining studies did not report this information [17,18,20,22,23]. Five studies (62.5%) used RMSSD as the vagal-related HRV index to guide training in participants allocated to HRV-guided training groups [17,19,20,23,24], one (12.5%) SD_1_ [21], and two (25%) HF, of which, one used the auto-regressive method to determine power spectral density [22] and another one used Fast-Fourier Transform [18]. Four studies (50%) used a single-day HRV value with a moving reference criterion [18,19,21,22] and four (50%) a rolling averaged HRV value with a fixed reference criterion, of which, three used a 7-day averaged HRV value [17,20,24] and one a 3-day averaged HRV value [23]. Out of the four studies that used a moving reference criterion, three used a 10-day averaged HRV value [19,21,22] and one used the single previous-day HRV value [18], while out of the four studies that used a fixed reference criterion, three updated the reference criterion once at the middle of the intervention [17,20,24] and one used the reference criterion captured at baseline throughout the entire intervention period [23]. Three studies (37.5%) calculated the reference criterion as mean − (1·SD) [19,21,22], three (37.5%) as mean ± (0.5·SD) [17,20,24], one (12.5%) used the 70% of the previous day as reference criterion [18], and one (12.5%) used the mean value measured at baseline [23].

### 3.3. Risk of Bias

Details of the author’s judgements for each source of bias, and the risk of bias assessment across studies can be found in Tables S1 and S2 (see Supplementary Materials, Section S2), respectively. The method of sequence generation and allocation concealment were not reported in the included randomized studies (87.5%), and one non-randomized study was also included (12.5%). Only one study carried out blinded assessments. Therefore, selection and detection biases were judged as unclear-high risk. Attrition bias was judged as low-high risk, while reporting bias was judged as low risk.

### 3.4. Outcomes

Assessment characteristics for measuring cardiac-vagal modulation, aerobic fitness parameters, and endurance performance, as well as outcome details are provided in Table 3. Regarding cardiac-vagal modulation, one study (12.5%) assessed HRR 1 min after an incremental maximal test, five studies (62.5%) reported resting vagal-related HRV indices, and four (50%) resting HR, both indices obtained in several positions. One study (12.5%) used RMSSD as the vagal-related HRV index [19], one (12.5%) used SD_1_ [21], two (25%) used HF [18,22], and one (12.5%) measured RMSSD and HF [23]. Four studies (50%) used averaged HR and HRV values [19,21,22,23] and one (12.5%) captured a single HRV value at pre-intervention and an averaged HRV value at post-intervention [18]. Three studies (37.5%) carried out assessments in the morning [18,21,22], one (12.5%) in the afternoon/evening before training [19], and one (12.5%) measured vagal-related HRV indices at night and in the morning [23]. Nonetheless, incomplete information was reported to calculate the SMD in those studies using vagal-related HRV measured in the morning. All the included studies allowed us to define 19 independent comparisons as follows: HRR 1 min (*n* = 1) [19], standing vagal-related HRV indices (*n* = 6) [18,19,21,22], standing HR (*n* = 5) [18,21,22], sitting vagal-related HRV indices (*n* = 1) [22], sitting HR (*n* = 1) [22], supine vagal-related HRV indices (*n* = 1) [18], supine HR (*n* = 1) [18], nocturnal vagal-related HRV indices (*n* = 2) [23], and nocturnal HR (*n* = 1) [23]. Regarding aerobic fitness parameters and endurance performance, all the included studies performed an incremental test until volitional exhaustion, of which, seven (87.5%) also performed ventilatory gas exchange assessments. Five studies (62.5%) carried out a sport-specific time trial for assessing endurance performance. All the included studies allowed us to define 32 independent comparisons between HRV-guided training and predefined training as follows: $\dot{V}$O_2_ max (*n* = 9) [17,18,20,21,22,23,24], $\dot{V}$O_2_ at VT2 (*n* = 1) [18], maximal aerobic capacity (*n* = 8) [17,19,20,21,22,23], aerobic capacity at VT2 (*n* = 5) [17,20,22,23,24], aerobic capacity at VT1 (*n* = 4) [17,20,23,24], and endurance performance (*n* = 5) [17,19,20,23,24]. As previously described, at least three analysis units should report each outcome measure to be pooled for meta-analysis. Otherwise, the results will be qualitatively discussed in the next section.

#### 3.4.1. Cardiac-Vagal Modulation

**Figure 2:**
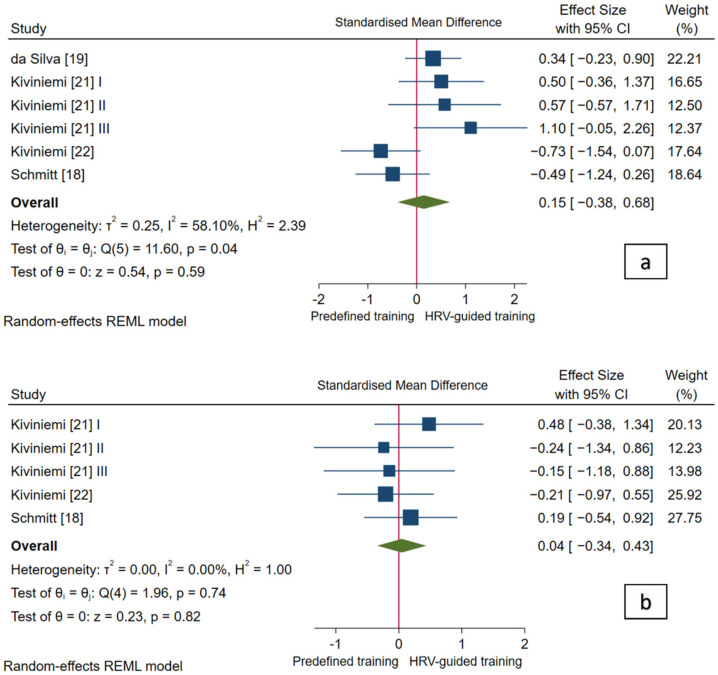
Forest plot of standardized mean difference indices for cardiac-vagal modulation: (**a**) standing vagal-related heart rate variability indices, and (**b**) standing heart rate. I, II, III refer analysis units from the same study.

**Figure 3:**
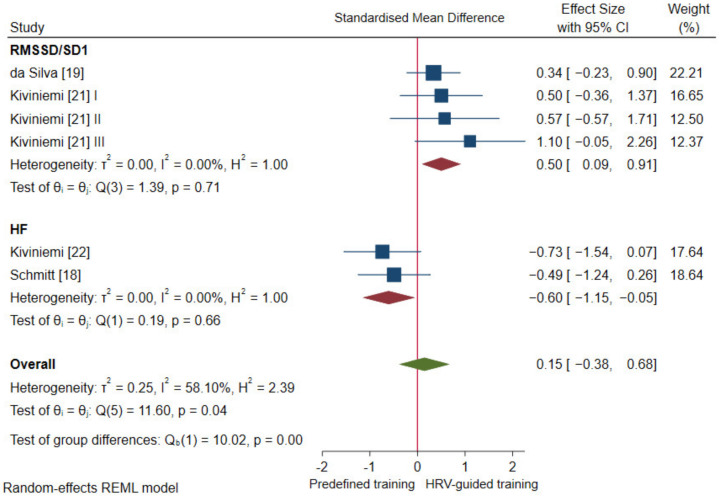
Test for subgroup comparisons for standing vagal-related HRV indices based on the HRV index used. I, II, III refer analysis units from the same study.

Pooled analysis revealed no statistically significant difference in standing vagal-related HRV indices (*p* = 0.59) and standing HR (*p* = 0.82) between HRV-guided training and predefined training, and the overall SMDs reached a trivial effect (SMD_+_ = 0.15 (95% CI = −0.38, 0.68), and SMD_+_ = 0.04 (95% CI = −0.34, 0.43), respectively; Figure 2). The heterogeneity test reached statistical significance (*p* = 0.04) and inconsistency was moderate (*I*^2^ = 58.1%) for standing vagal-related HRV indices, while the heterogeneity test did not reach statistical significance (*p* = 0.74) and no inconsistency was found (*I*^2^ = 0.0%) for standing HR. Therefore, analyses of the influence of methodological factors on the pooled findings for standing vagal-related HRV indices were carried out. Our subgroup analyses showed significant between-group heterogeneity for the vagal-related HRV index (i.e., RMSSD/SD_1_ and HF) (*p* < 0.01). There were greater increases in RMSSD/SD_1_ (SMD_+_ = 0.50 (95% CI = 0.09, 0.91)) and greater decrements in HF (SMD_+_ = −0.60 (95% CI = −1.15, −0.05)) after HRV-guided training compared to predefined training (see Figure 3). Subgroup analysis based on the HRV value (i.e., single and averaged HRV values) was not performed since none of the included studies used a single HRV value at pre- and post-intervention. Within-group heterogeneity, based on the vagal-related HRV index (i.e., RMSSD/SD_1_ and HF), was not found (*I*^2^ = 0%). Thus, the influence of participant and methodological approach characteristics on vagal-related HRV indices was not studied.

#### 3.4.2. Aerobic Fitness Parameters and Endurance Performance

**Figure 4:**
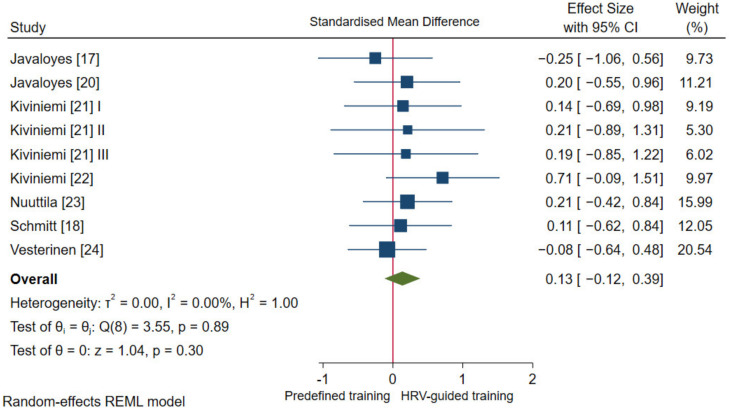
Forest plot of standardized mean difference indices for $\dot{V}$O_2_ max. I, II, III refer analysis units from the same study.

Pooled analysis revealed no statistically significant difference in $\dot{V}$O_2_ max (*p* = 0.30) between HRV-guided training and predefined training, and the overall SMD reached a trivial effect (SMD_+_ = 0.13 (95% CI = −0.12, 0.39); Figure 4). The heterogeneity test did not reach statistical significance (*p* = 0.89) and no inconsistency was found (*I*^2^ = 0.0%). Therefore, the influence of moderator variables on $\dot{V}$O_2_ max changes after HRV-guided training vs. predefined training was not analyzed.

**Figure 5:**
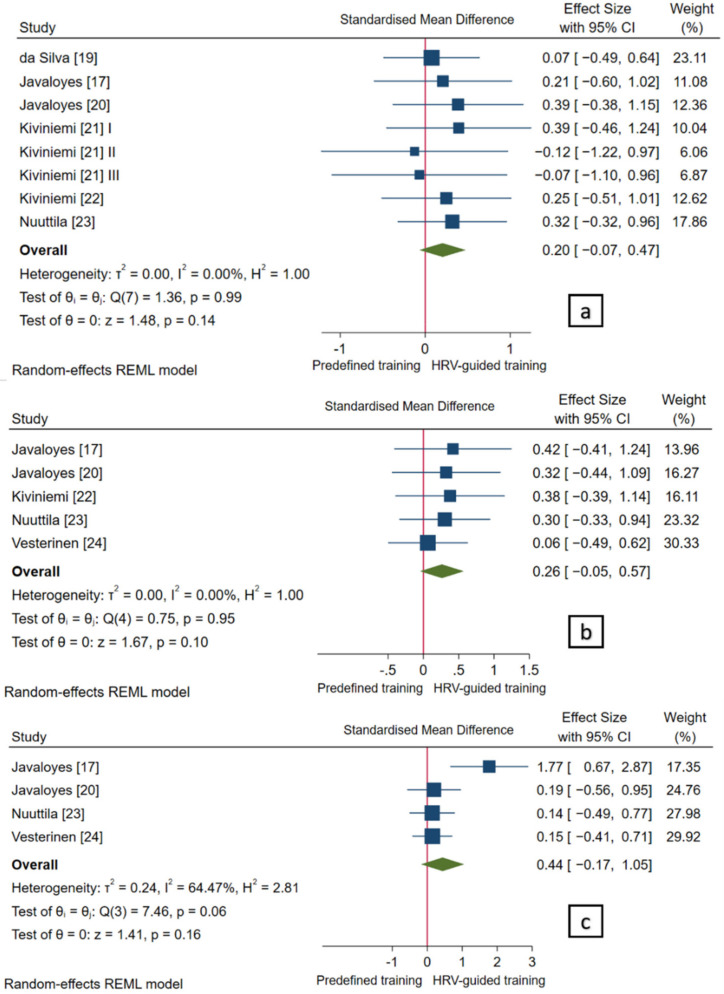
Forest plot of standardized mean difference indices for (**a**) maximal aerobic capacity, (**b**) aerobic capacity at second ventilatory threshold, and (**c**) aerobic capacity at first ventilatory threshold. I, II, III refer analysis units from the same study.

Pooled analyses showed no statistically significant differences in maximal aerobic capacity (*p* = 0.14), aerobic capacity at VT2 (*p* = 0.10), and aerobic capacity at VT1 (*p* = 0.16) between both training prescription methods. Nevertheless, the overall SMDs reached a small effect in favor of HRV-guided training (SMD_+_ = 0.20 (95% CI = −0.07, 0.47), SMD_+_ = 0.26 (95% CI = −0.05, 0.57), and SMD_+_ = 0.44 (95% CI = −0.17, 1.05), respectively; Figure 5) compared to predefined training. Heterogeneity tests did not reach statistical significance (*p* > 0.05) and no inconsistency was found (*I*^2^ = 0.0%) for maximal aerobic capacity and aerobic capacity at VT2, showing no influence of potential moderator characteristics on these variables. Despite the existence of a non-significant heterogeneity test (*p* = 0.06), inconsistency was moderate for aerobic capacity at VT1 (*I*^2^ = 64.5%). However, due to the low number of studies, the influence of potential moderator variables was not performed. 

**Figure 6:**
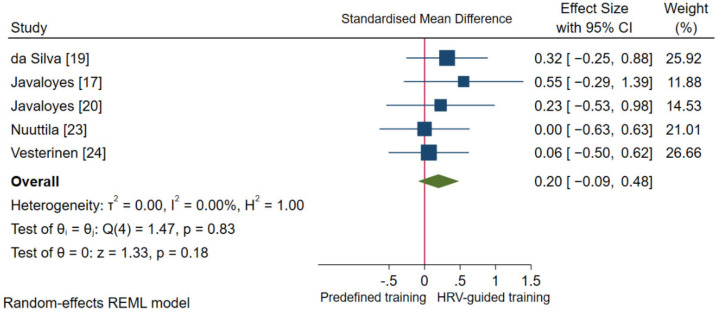
Forest plot of standardized mean difference indices for endurance performance.

Pooled analysis showed no statistically significant difference in endurance performance (*p* = 0.18) between HRV-guided training and predefined training. However, the overall SMD reached a small effect in favor of HRV-guided training (SMD_+_ = 0.20 (95% CI = −0.09, 0.48); Figure 6) compared to predefined training. The heterogeneity test was non-significant (*p* = 0.83), and no inconsistency was found (*I*^2^ = 0.0%). Therefore, the influence of moderator variables on endurance performance changes was not investigated.

### 3.5. Publication Bias

There was no evidence of asymmetry in the funnel plots for any of the analyzed variables and the trim-and-fill method imputed no ESs to symmetrize the funnel plots (see supplementary materials, Section S3, Figures S1–S7). Therefore, on a reasonable basis, publication bias can be discarded as a threat against the validity of our findings. 

### 3.6. Sensitivity Analysis

Our sensitivity analyses showed no influence of any individual study for cardiac-vagal modulation, $\dot{V}$O_2_ max, maximal aerobic capacity, aerobic capacity at VT2, and endurance performance. Nonetheless, the overall SMD and heterogeneity for aerobic capacity at VT1 diminished (from SMD_+_ = 0.44 (95% CI = −0.17, 1.05) to SMD_+_ = 0.16 (95% CI = −0.21, 0.52), and from 64.5% to 0.0%, respectively) after removing Javaloyes, Sarabia, Lamberts, Plews, Moya-Ramon [17].

## 4. Discussion

This systematic review with meta-analysis investigated the effects of HRV-guided training versus predefined training for improving cardiac-vagal modulation, aerobic fitness, and endurance performance in sedentary healthy people, physically active, and endurance-trained athletes. Results showed that the effect of training prescription style on cardiac-vagal activity was index-dependent, such that greater increases in RMSSD/SD_1_ were observed for HRV-guided training and vice-versa for HF. Our findings further showed that HRV-guided training was not significantly greater than predefined training for improving maximal aerobic capacity, aerobic capacity at VT2, and endurance performance, though small ESs consistently favored HRV-guided training. No heterogeneity was found for any aerobic fitness and performance parameters included in our pooled analyses. This indicates that there was no influence of potential moderator variables (e.g., baseline participant characteristics and methodological approach characteristics) on the difference between training prescription methods for improving these outcomes. 

This is the first systematic review with meta-analysis to investigate the effectiveness of HRV-guided training versus predefined training for enhancing cardiac-vagal modulation. Although pooled analyses showed no significant differences between training approaches, significant heterogeneity was observed for the vagal-related HRV index used to reflect autonomic adaptation (Figure 2). Follow-up subgroup analysis revealed that HRV-guided training was superior to predefined training for increasing RMSSD/SD_1_, whereas the opposite was found for HF (Figure 3). Certain methodological factors may account for the inconsistent responses among the vagal-related HRV indices. For instance, HF is more influenced by breathing rate than RMSSD/SD_1_ [52,53], but whether respiration was standardized in studies using HF was not disclosed [18,22]. Moreover, Schmitt, Willis, Fardel, Coulmy, Millet [18] compared single time-point HF values obtained pre-intervention with a 21-day averaged value obtained post-intervention. Isolated values inadequately represent autonomic status [12,54,55,56] and averaged values from such a lengthy follow-up are likely influenced by alterations in training (i.e., cessation, resumption, or variation not specified) and thus may not suitably reflect effects of the intervention [18]. The remaining studies used pre- and post-intervention RMSSD or SD_1_ values averaged across 3–7 days in accordance with recent findings [12,54,55,56,57,58]. Finally, previous studies have reported potential bias of spectral indices due to non-stationarities [59]. Thus, methodological factors from studies that used HF [18,22] may explain the heterogeneity found in our pooled analysis for vagal-related HRV indices. 

Most studies in our analysis recorded vagal-related HRV indices in the standing position, whilst two studies also included seated [22] and supine [18] measures (Table 3). A subgroup analysis based on the assessment position was not performed to avoid statistical dependence (i.e., inclusion of participants twice for multiple positions). The original rationale for adopting standing measures was to counteract the effects of parasympathetic saturation [22], commonly observed during traditional supine recordings [60]. This results in reduced HRV concurrent with reduced resting HR due to saturation of myocardial cholinergic receptors from parasympathetic predominance, reflecting a quadratic relationship between parasympathetic activity and HRV [61,62]. Thus, HRV-guided training prescription in such instances would be unmatched (i.e., low intensity or rest due to low HRV) with the true status of the autonomic nervous system (high parasympathetic activity). Orthostatic stress during standing provokes baroreflex-mediated cardiac-autonomic and hemodynamic adjustments to maintain cardiac output and overcome blood-pooling in the lower extremities. Accordingly, supine and standing positions represent distinct physiological conditions that have demonstrated varying timeframes of post-exercise HRV recovery [63]. In addition, daily standing RMSSD patterns are generally lower and more variable relative to supine values [58,64], and whether they are correlated (i.e., provide similar intra-individual HRV trends despite different absolute values) is unclear. Thus, it is possible that exercise prescription on the basis of daily HRV would vary depending on the recording position and potentially impact adaptations. One recent review paper identified standing measures as being more sensitive to changes in parasympathetic activity than other positions [56]. However, the optimal HRV assessment position for guiding daily training prescription and reflecting autonomic adaptation remains unclear. 

Though post-intervention improvements in HRV are of interest, responses observed amid training may be of similar or greater relevance. Several investigations and one case study reported greater reductions in vagal-mediated HRV relative to baseline throughout predefined training versus better maintenance of values with HRV-guided training [17,18,65]. Moreover, observational studies frequently report greater aerobic fitness improvements among individuals who exhibit higher and more stable vagal HRV values throughout predefined training [37,66,67,68,69,70,71]. Contrastingly, greater day-to-day fluctuations in HRV are often observed in fatigued athletes and can occur with [58,68,72] or without [66,73] purposeful overload. Importantly, acute reductions in training stress enables suppressed HRV to revert to baseline [58,72]. Thus, it seems that HRV responses associated with improved adaptation and greater health (i.e., higher and more stable values) may be intentionally facilitated by adjusting training based on HRV. This strategy may support adaptations by matching the training stimulus with the current adaptive state of the autonomic nervous system [74], and by limiting wear-and-tear from excessive training load [19,20,22]. To improve our understanding of how training approaches impact cardiac-autonomic activity, we encourage future comparison studies to report inter-group HRV trend characteristics (e.g., averages and coefficient of variation) from before, during, and after the intervention.

Regarding other HR-based indices, da Silva, Ferraro, Adamo, Machado [19] measured HRR 1 min post-maximal incremental running tests in sedentary females. Characteristics of the recovery such as position or standardization of respiratory rate were not reported. Greater improvements in HRR 1 min were observed for HRV-guided training; however current results were non-significant and underpowered. Previous studies have reported that HRR 1 min is a sensitive index for reflecting autonomic adaptation [75] and may carry clinically-relevant implications related to cardio-metabolic morbidity and mortality [76]. Thus, future studies should investigate whether HRV-guided training is superior to predefined training for enhancing HRR 1 min. Pooled findings for resting HR showed no differences between training methods with no heterogeneity for improving resting HR assessed in the standing position. Studies that were not pooled because resting HR measurements were performed in other positions (i.e., sitting or supine) [18,22] or times of the day (i.e., night) [23], also failed to show differences between HRV-guided training and predefined training for changing resting HR (see Table 3). 

No significant differences between training prescription methods were observed for improving aerobic fitness and endurance performance. These findings agree with Medellin Ruiz, Rubio-Arias, Clemente-Suarez, Ramos-Campo [27]. Albeit non-significant, our pooled analyses showed small ESs in favor of HRV-guided training for improving maximal aerobic capacity, aerobic capacity at VT2, and endurance performance versus predefined training. Unlike the current and previous findings [27,28], Granero-Gallegos, González-Quílez, Plews, Carrasco-Poyatos [25] reported a significant effect for $\dot{V}$O_2_ max favoring HRV-guided training. We noted that between-group comparisons to compare the effectiveness of both training prescription methods for improving $\dot{V}$O_2_ max was not reported, and the overall training effect result seems to be reported in their forest plot instead [25]. Therefore, the conclusion of this study should be considered with caution. Length of training intervention may help explain the small magnitude of the ES for HRV-guided training versus predefined training. The longest training intervention from studies included herein was 8-weeks (75% of studies). Short-term predefined endurance training programs (i.e., 6 to 10 weeks) enhance aerobic fitness and endurance performance in sedentary and endurance-trained individuals [77,78], with plateaus in $\dot{V}$O_2_ max often observed with longer-term training [79]. The short duration of the reviewed training interventions may help explain why ESs favoring HRV-guided training were only small in magnitude. Moreover, HRV-guided regulation of exercise volume and intensity over chronic training periods may support performance and fitness gains by limiting maladaptions. For example, fatigue-related decrements in HRV left unabated may reflect heightened risk of infection, overuse, or overreaching [12,80]. Thus, future research should determine if longitudinal HRV-guided training offers any direct or indirect fitness or performance advantages over predefined training. 

No heterogeneity was found when comparing training methods for enhancing aerobic fitness or endurance performance, despite inclusion of samples varying in training status and history (i.e., sedentary to well-trained), age, and sex. These descriptive characteristics often impact responsiveness to training interventions [81]. Nevertheless, we noted that only da Silva, Ferraro, Adamo, Machado [19] included exclusively sedentary people. In agreement with our findings, Kiviniemi, Hautala, Kinnunen, Nissilä, Virtanen, Karjalainen, Tulppo [21] found no sex-related differences in response to HRV-guided training compared to predefined training. Thus, our findings apply to healthy adult males and females between the ages of 22 to 39 years. Future studies should compare HRV-guided versus predefined training for improving aerobic fitness and endurance performance in young, elderly, and clinical populations. Initial evidence among the latter suggests that HRV-guided training may be more effective than predefined training in cardiac-rehabilitation [82].

Our systematic review showed between-study variability in the use of the daily versus rolling averaged HRV values and in the fixed versus rolling reference criteria used to guide prescription in the HRV-guided training group. All the studies that used a rolling averaged HRV value also used a fixed reference criterion (i.e., 3- or 4-week baseline period), which was maintained throughout the training [23] or updated mid-training period [17,20,24]. Studies that used a single day HRV value used a moving reference criterion (i.e., 10 values) [19,21,22]. The use of rolling averaged HRV values results in less frequent training modifications relative to using daily values. Additionally, rolling reference criteria reflect current responses while fixed values reflect the initial baseline profile. It remains unclear which approach may be superior for improving training adaptations. Future studies are therefore needed to compare HRV-guided training methodologies to further establish best practices.

Changes in aerobic fitness and endurance performance following predefined training may be more heterogeneous [6,83] than changes observed following HRV-guided training. Javaloyes, Sarabia, Lamberts, Plews, Moya-Ramon [17] found a post-training performance decrement in only one cyclist allocated to the HRV-guided training group (14.3%) versus three athletes in the predefined training group (37.5%). Kiviniemi, Hautala, Kinnunen, Tulppo [22] reported a more homogeneous positive response in maximal running velocity for the HRV-guided training group. Similarly, $\dot{V}$O_2_ max decreased in only one runner after HRV-guided training (11.1%) versus four runners after predefined training (50.0%). Nevertheless, the low number of athletes included in these studies limits the scope of the findings. Therefore, future studies should analyze and report individual participant changes to investigate heterogeneity in adaptations to the training prescription method used.

This systematic review with meta-analysis is the first to investigate the effects of HRV-guided versus predefined training on cardiac-vagal modulation in sedentary healthy people, physically active, and endurance-trained athletes. Consideration of methodological factors in regard to HRV index selection, recording position, and approaches for establishing baseline reference values and daily changes (i.e., fixed or rolling HRV averages) are key strengths of the current study. However, a limited number of overall investigations, in addition to inconsistent methodological approaches, limit our ability to perform sufficient subgroup analyses to make strong conclusions. Similarly, the low number of studies included in the subgroup analysis for vagal-related HRV index selection limits the scope of our findings. Limitations notwithstanding, our review identified numerous unresolved research questions pertaining to methodological approaches to HRV-guided training that warrant further investigation. 

## 5. Conclusions

Our results generated a novel insight regarding the effects of HRV-guided training on cardiac-vagal activity and adds clarification about its impact on fitness and performance relative to predefined training. HRV-guided training demonstrated a small advantage over predefined training for improving vagal-mediated HRV (i.e., RMSSD/SD_1_) measured in standing position when averaged between 3–7 days. Similar findings were not observed for HF, possibly due to methodological factors related to standardization of respiratory rate and use of insufficient (i.e., isolated) or excessive (i.e., 3-week) periods of comparison. Effects on supine and seated HRV and post-exercise HRR were indeterminate. Qualitative review of available data further indicated that HRV-guided training facilitates greater maintenance of HRV values throughout an intervention relative to predefined training. By design, this training method prevents sustained decrements in HRV that may occur with excess training and fatigue, and which are often associated with smaller or negative changes in fitness markers. HRV-guided training did not produce significantly greater fitness and performance outcomes relative to pre-planned training, though ESs that were small in magnitude consistently favored HRV-guided training. Qualitative reviews of studies reporting individual changes in fitness and performance indicate that responses were more homogenous among HRV-guided training groups with fewer negative responders relative to predefined training. Lastly, despite our observation of high heterogeneity for methodological characteristics among studies, no inconsistency was found for any of the aerobic fitness and endurance performance parameters analyzed. In sum, HRV-guided training is an accessible individualized exercise prescription strategy that may be more effective than predefined training for maintaining and improving vagal-mediated HRV, with less likelihood of negative responses. However, if HRV-guided training is superior to predefined training for producing group-level improvements in fitness and performance, current data suggest it is only by a small margin.

## Figures and Tables

**Table 1:** Study and participant characteristics.

Study (Author, Year)	Training Group	Study Characteristics		Participant Characteristics	
		Country; Study Design; Journal	Sample Size; Men Percentage	Age; Weight; $\dot{V}$O_2_ Max	Athletic Status; Sport (If Applicable)
da Silva et al. [19] 2019	HRV-G	Brazil; randomized controlled trial; J Strength Cond Res	15; 0%	25.8 ± 3.1 years; 62.9 ± 10.3 kg; NR	Sedentary; NA
	PRED-G		15; 0%	27.7 ± 3.6 years; 61.3 ± 10.5 kg; NR	
Javaloyes et al. [17] 2020	HRV-G	Spain; non-randomized controlled trial; J Strength Cond Res	7; 100%	28.1 ± 13.2 years; 73.8 ± 4.6 kg; 58.9 ± 5.6 mL·kg^−1^·min^−1^	Well-trained; cyclists
	PRED-G		8; 100%	30.8 ± 10.5 years; 72.6 ± 10.4 kg; 59.0 ± 6.2 mL·kg^−1^·min^−1^	
Javaloyes et al. [20] 2019	HRV-G	Spain; randomized controlled trial; Int J Sport Physiol Perform	9; 100%	39.2 ± 5.3 years; 76.9 ± 12.5 kg; 55.0 ± 7.6 mL·kg^−1^·min^−1^	Well-trained; cyclists
	PRED-G		8; 100%	37.6 ± 7.1 years; 78.7 ± 11.7 kg; 52.2 ± 6.5 mL·kg^−1^·min^−1^	
Kiviniemi et al. [21] 2010	HRV-G	Finland; randomized controlled trial; Med Sci Sports Exerc	7; 100%	35.0 ± 4.0 years; 82.0 ± 9.0 kg; 50.0 ± 6.0 mL·kg^−1^·min^−1^	Physically active; NA
	PRED-G		7; 100%	37.0 ± 3.0 years; 81.0 ± 14.0 kg; 50.0 ± 7.0 mL·kg^−1^·min^−1^	
	HRV-G		7; 0%	33.0 ± 4.0 years; 64.0 ± 5.0 kg; 36.0 ± 4.0 mL·kg^−1^·min^−1^	
	HRV-G		10; 0%	35.0 ± 4.0 years; 64.0 ± 9.0 kg; 37.0 ± 5.0 mL·kg^−1^·min^−1^	
	PRED-G		7; 0%	34.0 ± 4.0 years; 67.0 ± 6.0 kg; 35.0 ± 5.0 mL·kg^−1^·min^−1^	
Kiviniemi et al. [22] 2007	HRV-G	Finland; randomized controlled trial; Eur J Appl Physiol	9; 100%	31.0 ± 6.0 years; 80.0 ± 8.0 kg; 56.0 ± 4.0 mL·kg^−1^·min^−1^	Recreationally trained; runners
	PRED-G		8; 100%	32.0 ± 5.0 years; 78.0 ± 8.0 kg; 54.0 ± 4.0 mL·kg^−1^·min^−1^	
Nuuttila et al. [23] 2017	HRV-G	Finland; randomized controlled trial; Int J Sports Med	13; 100%	29.0 ± 4.0 years; 76.4 ± 9.4 kg; 53.6 ± 4.2 mL·kg^−1^·min^−1^	Recreationally trained; endurance athletes
	PRED-G		11; 100%	31.5 ± 5.0 years; 74.0 ± 5.7 kg; 54.2 ± 4.1 mL·kg^−1^·min^−1^	
Schmitt et al. [18] 2018	HRV-G	France, randomized controlled trial; Eur J Appl Physiol	9; 78%	22.4 ± 3.9 years; 65.5 ± 7.2 kg; 66.7 ± 5.9 mL·kg^−1^·min^−1^	Highly trained; cross-country and nordic-skiers
	PRED-G		9; 67%	22.6 ± 3.2 years; 66.7 ± 10.1 kg; 63.7 ± 4.4 mL·kg^−1^·min^−1^	
Vesterinen et al. [24] 2016	HRV-G	Finland; randomized controlled trial; Me Sci Sports Exerc	20; NR *	34.5 ± 7.5 years ^#^; NR; 54.4 ± 6.2 mL·kg^−1^·min^−1^	Recreationally trained; runners
	PRED-G		20; NR *	34.5 ± 7.5 years ^#^; NR; 53.0 ± 5.8 mL·kg^−1^·min^−1^	

HRV-G, heart rate variability guided training group; *NA*, non-applicable; *NR*, no reported; *PRED-G*, predefined training group; $\dot{V}$O_2_ max, maximal oxygen uptake. Data are reported as mean ± standard deviation, unless otherwise is stated; * 20 males and 20 females were allocated at pre-intervention; ^#^ Based on all participants.

**Table 2:** Intervention and methodological approach characteristics.

Study (Author)	Intervention Characteristics	Methodological Approach Characteristics		
	Type of Exercise; Length; Training Frequency	Device; Time of Day; Stabilization Period (Min); Recording Posture (Length) *; Breathing Control	HRV Index; Single Day vs. Averaged; Number of Averaged Values	Fixed vs. Moving; Number of Averaged Values; Range Used
da Silva et al. [19]	Endurance training; 8 weeks; 3 days a week	Polar RS800cx; afternoon/evening; yes (2 min); standing (3 min); no (spontaneous)	RMSSD; single day; NA	Moving; 5 up to 10 values; mean − (1·SD)
Javaloyes et al. [17]	Endurance training; 8 weeks; NA (habitual training volume)	HRV4training app; morning; yes (30 s); supine (1 min); NR	RMSSD; averaged; 7 values	Fixed ^$^; 28 values; mean ± (0.5·SD)
Javaloyes et al. [20]	Endurance training; 8 weeks; NA (habitual training volume)	Polar H7 strap; morning; yes (30 s); supine (1 min); NR	RMSSD; averaged; 7 values	Fixed ^$^; 28 values; mean ± (0.5·SD)
Kiviniemi et al. [21]	Endurance training; 8 weeks; at least 5 days a week ^#^	Polar RS800; morning; yes (2 min); standing (3 min); no (spontaneous)	SD_1_; single day; NA	Moving; 7 up to 10 values; mean − (1·SD)
Kiviniemi et al. [22]	Endurance training; 4 weeks; 6 days a week ^#^	Polar S180i; morning; yes (5 min); standing (5 min); NR	HF (auto-regressive method); single day; NA	Moving; 10 values; mean − (1·SD)
Nuuttila et al. [23]	Endurance and strength training; 8 weeks; 6 days a week ^#^	Garmin 920XT; morning; supine (3 min); yes (until heart rate became steady); NR	RMSSD; averaged; 3 values	Fixed; 21 values; Mean
Schmitt et al. [18]	NR; 2 weeks; NR	Suunto; morning; yes (3 in supine and 1 in standing); supine and standing (5 + 5 min); NR	HF (Fast-Fourier Transform); single day; NA	Moving; 1 value; 70% of the previous day
Vesterinen et al. [24]	Endurance and strength training; 8 weeks; 2–4 days a week ^#^	Omegawave Pro Mobile System; morning; no stabilization; supine (4 min); no (spontaneous)	RMSSD; averaged; 7 values	Fixed ^$^; 28 values; mean ± (0.5·SD)

HF, high frequency; *HRV*, heart rate variability; *NA*, non-applicable; *NR*, no reported; *RMSSD*, root-mean-square difference of successive normal R-R intervals; *SD*, standard deviation; *SD_1_*, standard deviation of instantaneous beat-to-beat R-R interval variability. ^#^ Only in the predefined training group; * analyzed period; ^$^ Fixed reference criterion was updated.

**Table 3:** Assessment characteristics and outcome details.

Study (Author); N (HRV-G/PRED-G)	Aerobic Fitness Parameters and Endurance Performance		Cardiac-Vagal Modulation	
	Assessment Characteristics	Parameter Assessed: SMD (95% CI)	Assessment Characteristics	Parameter Assessed: SMD (95% CI)
da Silva et al. [19] N (15/15)	Incremental running test until volitional exhaustion	Maximal velocity (MAC): 0.07 (−0.49, 0.63)	Incremental maximal running test; recovery characteristics no reported	^$^ HRR 1 min: 0.20 (−0.36, 0.77)
	5 km running performance	Time (EP): 0.31 (−0.26, 0.87)	3-day averaged values measured in standing position in the afternoon/evening	Standing RMSSD: 0.34 (−0.23, 0.90)
Javaloyes et al. [17] N (7/8)	Incremental cardiopulmonary cycling test until volitional exhaustion	$\dot{V}$O_2_ max: −0.25 (−1.06, 0.56)		
		Maximal PO (MAC): 0.21 (−0.60, 1.02)		
		PO at VT2 (AC_VT2): 0.42 (−0.41, 1.24)		
		PO at VT1 (AC_VT1): 1.77 (0.67, 2.87)		
	40 min all-out time trial	Mean PO (EP): 0.55 (−0.29, 1.39)		
Javaloyes et al. [20] N (9/8)	Incremental cardiopulmonary cycling test until volitional exhaustion	$\dot{V}$O_2_ max: 0.20 (−0.55, 0.96)		
		Maximal PO (MAC): 0.39 (−0.38, 1.15)		
		PO at VT2 (AC_VT2): 0.32 (−0.44, 1.09)		
		PO at VT1 (AC_VT1): 0.19 (−0.56, 0.95)		
	40 min all-out time trial	Mean PO (EP): 0.23 (−0.53, 0.98)		
Kiviniemi et al. [21] I N (7/7)	Incremental cardiopulmonary cycling test until volitional exhaustion	$\dot{V}$O_2_ max: 0.14 (−0.69, 0.98)	7-day averaged values measured in standing position in the morning	Standing SD_1_: 0.50 (−0.36, 1.37)
		Maximal PO (MAC): 0.39 (−0.46, 1.24)		Standing HR: 0.48 (−0.38, 1.34)
Kiviniemi et al. [21] II N (7/3)		$\dot{V}$O_2_ max: 0.21 (−0.89, 1.31)		Standing SD_1_: 0.57 (−0.57, 1.71)
		Maximal PO (MAC): −0.12 (−1.22, 0.97)		Standing HR: −0.24 (−1.34, 0.86)
Kiviniemi et al. [21] III N (10/3)		$\dot{V}$O_2_ max: 0.19 (−0.85, 1.22)		Standing SD_1_: 1.10 (−0.05, 2.26)
		Maximal PO (MAC): −0.07 (−1.10, 0.96)		Standing HR: −0.15 (−1.18, 0.88)
Kiviniemi et al. [22] N (9/8)	Incremental cardiopulmonary running test until volitional exhaustion	$\dot{V}$O_2_ max: 0.71 (−0.09, 1.51)	3-day averaged values measured in sitting and standing position in the morning	^$^ Sitting HF: 0.66 (−0.14, 1.45)
		Maximal velocity (MAC): 0.25 (−0.51, 1.01)		^$^ Sitting HR: 0.00 (−0.75, 0.75)
		Velocity at VT2 (AC_VT2): 0.38 (−0.39, 1.14)		Standing HF: −0.73 (−1.54, 0.07)
				Standing HR: −0.21 (−0.97, 0.55)
Nuuttila et al. [23] N (13/11)	Incremental cardiopulmonary running test until volitional exhaustion	$\dot{V}$O_2_ max: 0.21 (−0.42, 0.84)	Averaged 3-control weeks (11 days, measured every other night) and averaged last training week (4 days, measured every other night)	^$^ Night RMSSD: −0.05 (−0.68, 0.58)
		Maximal velocity (MAC): 0.32 (−0.32, 0.96)		^$^ Night HF: 0.10 (−0.53, 0.73)
		Velocity at VT2 (AC_VT2): 0.30 (−0.33, 0.94)		^$^ Night HR: 0.14 (−0.49, 0.77)
		Velocity at VT1 (AC_VT1): 0.14 (−0.49, 0.77)	21-day averaged (pre-intervention) and 7-day averaged (post-intervention) measured in supine position the morning	No reported
	3 km running performance	Time (EP): 0.00 (−0.63, 0.63)		
Schmitt et al. [18] N (9/9)	Incremental cardiopulmonary running test until volitional exhaustion	$\dot{V}$O_2_ max: 0.11 (−0.62, 0.84)	Single day (pre-intervention) and 21-day averaged (post-intervention) values measured in supine and standing position in the morning	^$^ Supine HF: −0.17 (−0.90, 0.57)
		^$^$\dot{V}$O_2_ at VT2: 0.24 (−0.49, 0.98)		^$^ Supine HR: 0.44 (−0.31, 1.18)
				Standing HF: −0.49 (−1.24, 0.26)
				Standing HR: 0.19 (−0.55, 0.92)
Vesterinen et al. [24] N (13/18)	Incremental cardiopulmonary running test until volitional exhaustion	$\dot{V}$O_2_ max: −0.08 (−0.64, 0.48)		
		Velocity at VT2 (AC_VT2): 0.06 (−0.49, 0.62)		
		Velocity at VT1 (AC_VT1): 0.15 (−0.41, 0.71)		
	3 km running performance	Mean velocity (EP): 0.06 (−0.50, 0.62)		

AC_VT1, aerobic capacity at first ventilatory threshold; *AC_VT2*, aerobic capacity at second ventilatory threshold; *EC*, endurance capacity; *EP*, endurance performance; *HF*, high frequency; *HR*, heart rate; *HRR 1 min*, heart rate recovery 1 min; *HRV-G*, heart rate variability guided training group; *MAC*, maximal aerobic capacity, *N*, number of participants included to calculate SMD; *PO*, power output; *PRED-G*, predefined training group; *RMSSD*, root-mean-square difference of successive normal R-R intervals; *SD_1_*, standard deviation of instantaneous beat-to-beat R-R interval variability; *SMD*, standardized mean difference; $\dot{V}$O_2_, oxygen uptake; *VT1*, first ventilatory threshold; *VT2*, second ventilatory threshold. ^$^ Excluded from meta-analysis as the minimal number of studies needed to perform pooled analyses was not reached; I, II, III refer analysis units from the same study.

## Data Availability

The dataset generated from the current study are available from the corresponding author on reasonable request.

## Supplementary Materials

The following are available online at https://www.mdpi.com/article/10.3390/ijerph181910299/s1, Section S1: Electronic database search, Section S2: Table S1. Risk of bias assessment criteria, Table S2. Risk of bias across studies, Section S3: Figure S1. Funnel plot with trim-and-fill method for standing vagal-related heart rate variability indices, Figure S2. Funnel plot with trim-and-fill method for standing heart rate, Figure S3. Funnel plot with trim-and-fill method for $\dot{V}$O_2_ max, Figure S4. Funnel plot with trim-and-fill method for maximal aerobic capacity, Figure S5. Funnel plot with trim-and-fill method for aerobic capacity at second ventilatory threshold, Figure S6. Funnel plot with trim-and-fill method for aerobic capacity at first ventilatory threshold, Figure S7. Funnel plot with trim-and-fill method for endurance performance.
