# Supplementary material for: Heart Rate Variability-Guided Training for Enhancing Cardiac-Vagal Modulation, Aerobic Fitness, and Endurance Performance: A Methodological Systematic Review with Meta-Analysis
Source: Int J Environ Res Public Health. 2021 Sep 29;18(19):10299. doi: 10.3390/ijerph181910299 (PMC8507742; doi:10.3390/ijerph181910299)
Supplement: Supplementary file 1 [file ijerph-18-10299-s001.zip › ijerph-1385259-supplementary.pdf]

Supplementary materials

Section 1: Electronic database search

- Participants. Healthy OR active OR sedentary OR trained OR untrained OR recreational OR fit OR young OR runn* OR swimm* OR cycli* OR triathl* OR athlete* OR rower* OR cross-countr* OR canoeist* OR kayak* OR skier* OR endurance

- Interventions. "Heart rate variability" OR HRV OR "HR variability" OR "training guided" OR "prescription guided" OR "individually guided" OR "individualized recovery"

- Comparison. Traditional OR predefined OR standard* OR periodization OR predetermined OR "fixed recovery"

Section 2

Table S1. Risk of bias assessment criteria.

**Reference: Da Silva et al. [19]**		
Bias	Authors' judgements	Support for judgement
Random sequence generation (selection bias)	Unclear risk	States participants were "randomly assigned". No further details given
Allocation concealment (selection bias)	Unclear risk	No mention of allocation concealment
Blinding of outcome assessment (detection bias)	High risk	Quote: "This is a non-blinded, randomised controlled trial"
Incomplete outcome data (attrition bias)	Low risk	Less than 20% missing outcome data, losses balanced, and reasons reported
Selective reporting (reporting bias)	Low risk	All pre-specified outcomes in the method section were reported
**Reference: Javaloyes et al. [17]**		
Bias	Authors' judgements	Support for judgement
Random sequence generation (selection bias)	High risk	Non-randomised controlled study
Allocation concealment (selection bias)	High risk	Non-randomised controlled study
Blinding of outcome assessment (detection bias)	High risk	Quote: "This is a non-blinded, controlled trial"
Incomplete outcome data (attrition bias)	High risk	More than 20% missing outcome data, losses balanced, and reasons reported
Selective reporting (reporting bias)	Low risk	All pre-specified outcomes in the method section were reported
**Reference: Javaloyes et al. [20]**		
Bias	Authors' judgements	Support for judgement
Random sequence generation (selection bias)	Unclear risk	States participants were "randomly assigned". No further details given
Allocation concealment (selection bias)	Unclear risk	No mention of allocation concealment
Blinding of outcome assessment (detection bias)	Unclear risk	No mention of outcome assessment blinding
Incomplete outcome data (attrition bias)	Unclear risk	Initial and final sample sizes were not reported
Selective reporting (reporting bias)	Low risk	All pre-specified outcomes in the method section were reported
**Reference: Kiviniemi et al. [21]**		
Bias	Authors' judgements	Support for judgement
Random sequence generation (selection bias)	Unclear risk	States participants were "randomised into". No further details given
Allocation concealment (selection bias)	Unclear risk	No mention of allocation concealment
Blinding of outcome assessment (detection bias)	Unclear risk	No mention of outcome assessment blinding
Incomplete outcome data (attrition bias)	Low risk	Less than 20% missing outcome data, losses balanced, and reasons reported
Selective reporting (reporting bias)	Low risk	All pre-specified outcomes in the method section were reported
**Reference: Kiviniemi et al. [22]**		
Bias	Authors' judgements	Support for judgement
Random sequence generation (selection bias)	Unclear risk	States participants were "randomised into". No further details given
Allocation concealment (selection bias)	Unclear risk	No mention of allocation concealment

Blinding of outcome assessment (detection bias)	Low risk	Quote: "Ventilatory threshold was analysed blindly from the…"
Incomplete outcome data (attrition bias)	Low risk	Less than 20% missing outcome data, losses balanced, and reasons reported
Selective reporting (reporting bias)	Low risk	All pre-specified outcomes in the method section were reported
**Reference: Nuuttila et al. [23]**		
Bias	Authors' judgements	Support for judgement
Random sequence generation (selection bias)	Unclear risk	States participants were "randomly assigned". No further details given
Allocation concealment (selection bias)	Unclear risk	No mention of allocation concealment
Blinding of outcome assessment (detection bias)	Unclear risk	No mention of outcome assessment blinding
Incomplete outcome data (attrition bias)	High risk	More than 20% missing outcome data and losses were reported regardless of the allocated groups
Selective reporting (reporting bias)	Low risk	All pre-specified outcomes in the method section were reported
**Reference: Schmitt et al. [18]**		
Bias	Authors' judgements	Support for judgement
Random sequence generation (selection bias)	Unclear risk	States participants were "randomly matched". No further details given
Allocation concealment (selection bias)	Unclear risk	No mention of allocation concealment
Blinding of outcome assessment (detection bias)	Unclear risk	No mention of outcome assessment blinding
Incomplete outcome data (attrition bias)	Unclear risk	Initial and final sample sizes were not reported
Selective reporting (reporting bias)	Low risk	All pre-specified outcomes in the method section were reported
**Reference: Vesterinen et al. [24]**		
Bias	Authors' judgements	Support for judgement
Random sequence generation (selection bias)	Unclear risk	States participants were "randomised into". No further details given
Allocation concealment (selection bias)	Unclear risk	No mention of allocation concealment
Blinding of outcome assessment (detection bias)	Unclear risk	No mention of outcome assessment blinding
Incomplete outcome data (attrition bias)	High risk	More than 20% missing outcome data and losses were reported regardless of the allocated groups
Selective reporting (reporting bias)	Low risk	All pre-specified outcomes in the method section were reported

Table S2. Risk of bias across studies.

**Risk of bias item**	**Low risk n/N (%)**	**Unclear risk n/N (%)**	**High risk n/N (%)**
Random sequence generation (selection bias)	0/8 (0)	7/8 (87.5)	1/8 (12.5)
Allocation concealment (selection bias)	0/8 (0)	7/8 (87.5)	1/8 (12.5)
Blinding of outcome assessment (detection bias)	1/8 (12.5)	5/8 (62.5)	2/8 (25)
Incomplete outcome data (attrition bias)	3/8 (37.5)	2/8 (25)	3/8 (37.5)
Selective reporting (reporting bias)	0/8 (0)	0/8 (0)	8/8 (100)

Section 3: Publication bias assessment

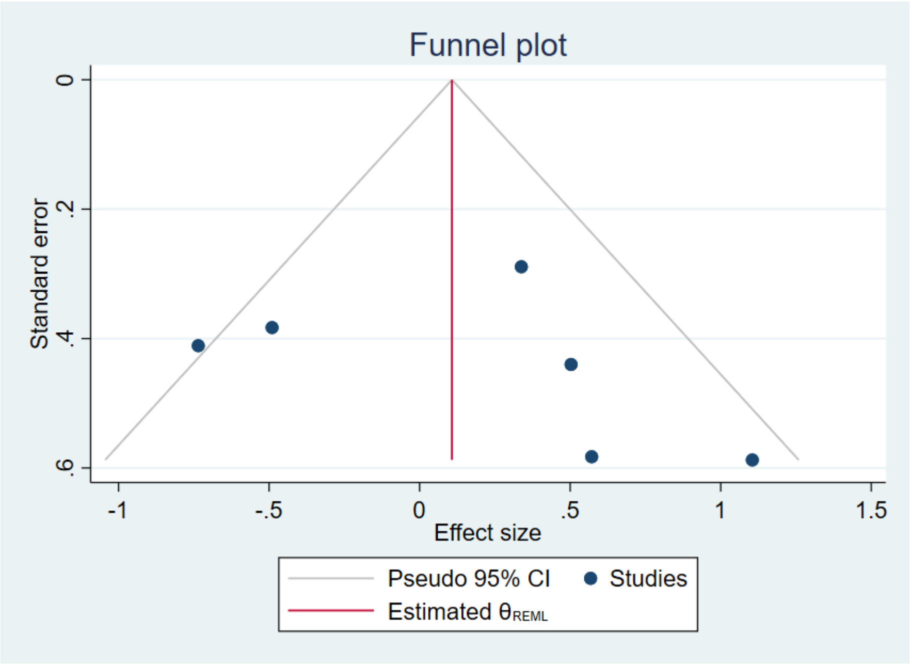

Figure S1. Funnel plot with trim-and-fill method for standing vagal-related heart rate variability indices

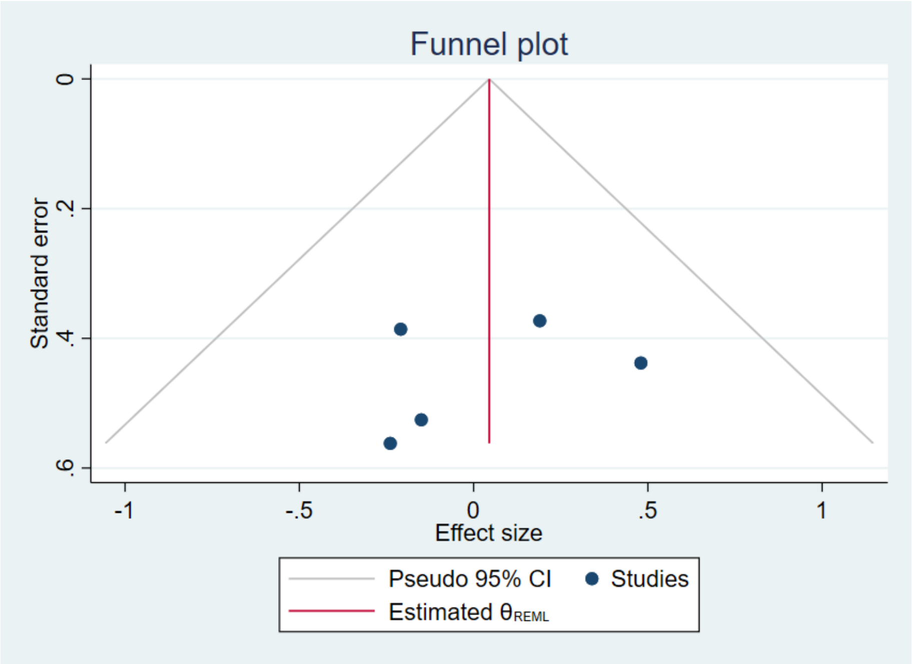

Figure S2. Funnel plot with trim-and-fill method for standing heart rate

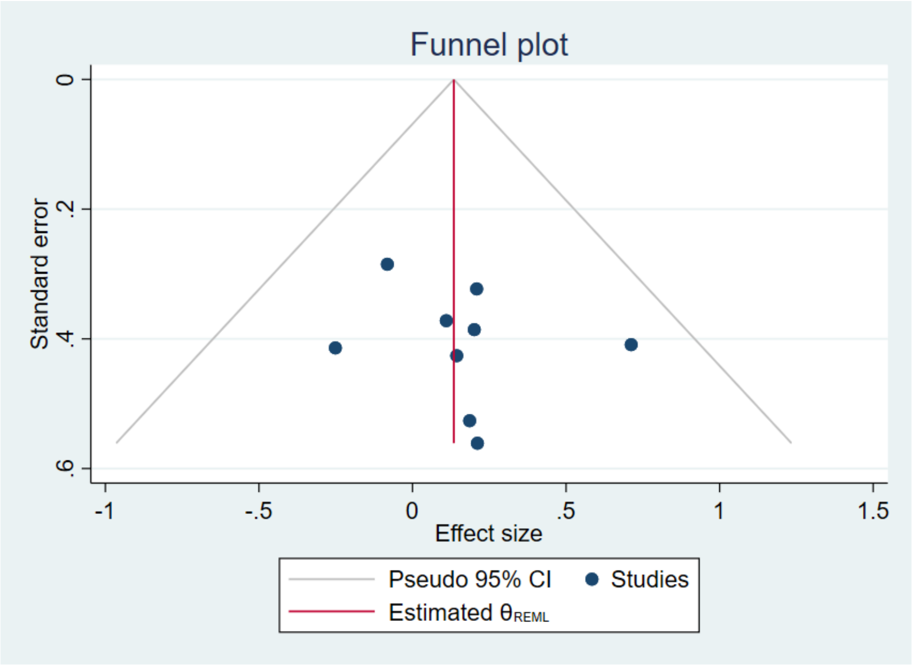

Figure S3. Funnel plot with trim-and-fill method for V̇O2 max

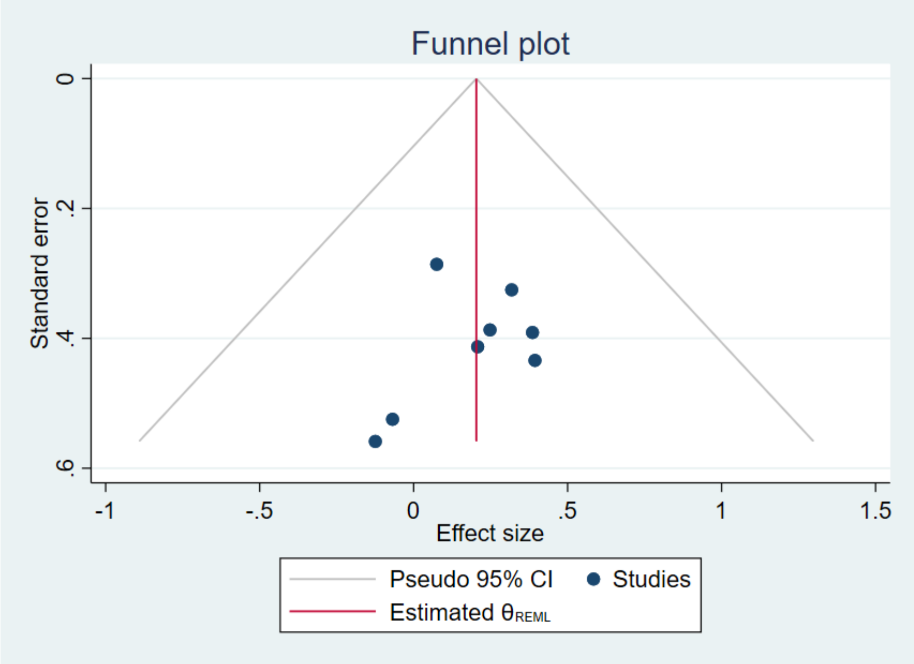

Figure S4. Funnel plot with trim-and-fill method for maximal aerobic capacity

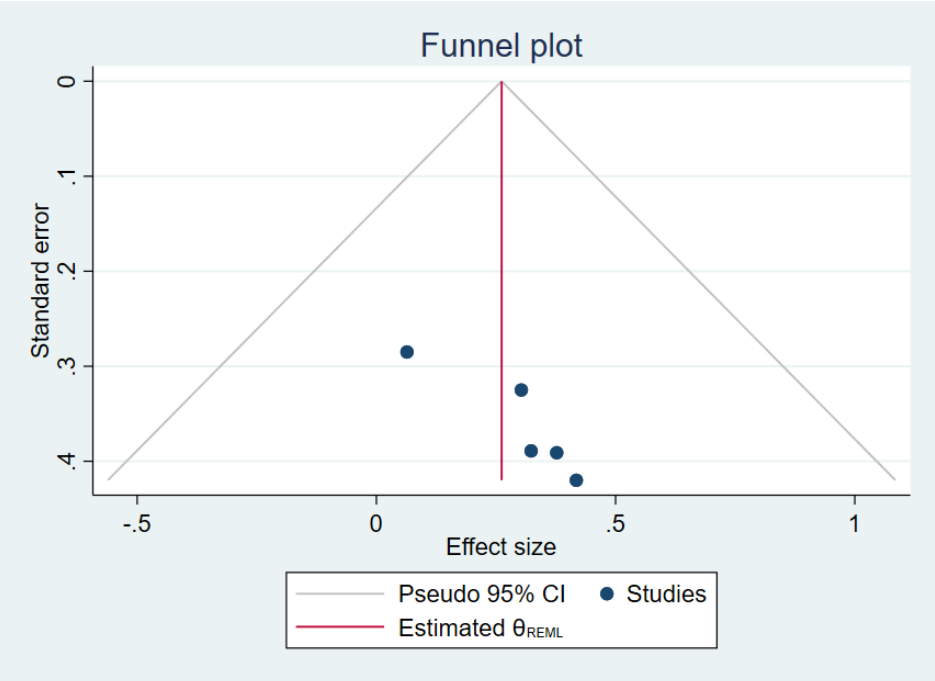

Figure S5. Funnel plot with trim-and-fill method for aerobic capacity at second ventilatory threshold

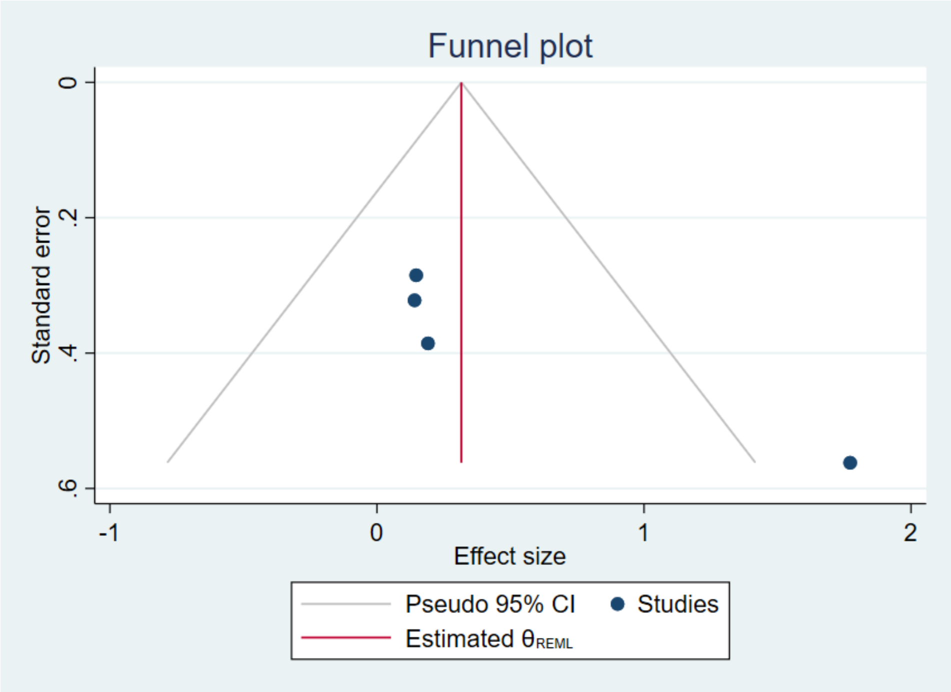

Figure S6. Funnel plot with trim-and-fill method for aerobic capacity at first ventilatory threshold

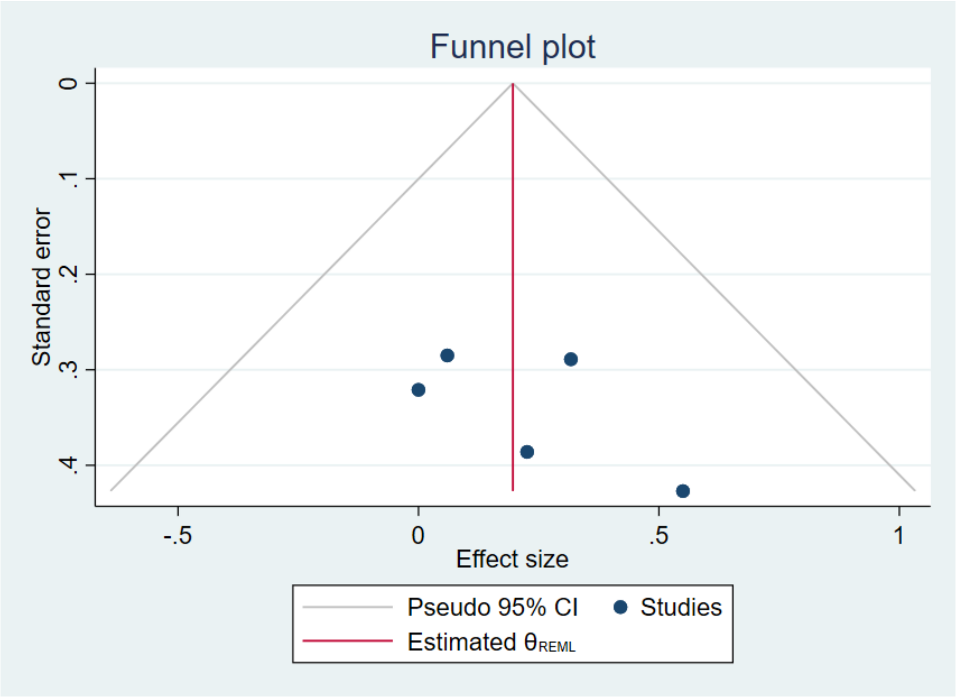

Figure S7. Funnel plot with trim-and-fill method for endurance performance
